# Myocardial perfusion scintigraphy: interpretation of a normal scan

**DOI:** 10.1007/s12471-014-0541-0

**Published:** 2014-03-06

**Authors:** P. Knaapen

**Affiliations:** Department of Cardiology, VU University Medical Center, De Boelelaan 1117, 1081 HV Amsterdam, the Netherlands

**Fig. 1:**
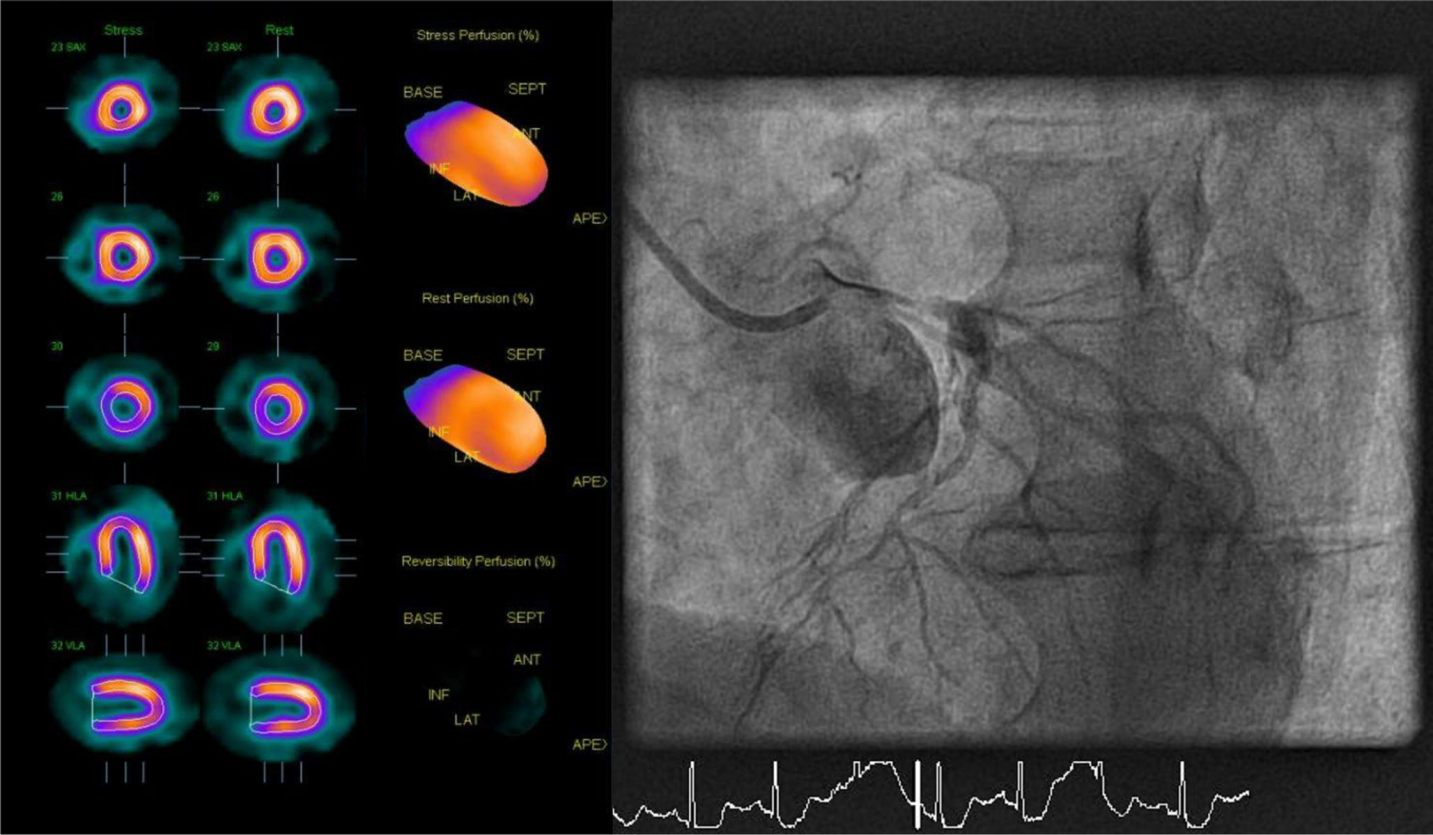
A 73-year-old male with atypical angina without cardiovascular risk factors and no prior cardiac history was evaluated for coronary artery disease with ^99m^Tc-sestamibi single photon emission computed tomography (SPECT) during rest and vasodilator stress (adenosine). Tracer distribution was homogenous during rest and stress, and the visual and automated grading of the images yielded a normal test result. Given the intermediate-to-high pre-test likelihood of disease and persistent symptoms, the patient was referred for invasive coronary angiography. Upon engagement of the left main coronary artery, the catheter wedged the artery and the patient became ischaemic and hypotensive. Subselective angiography displayed a subtotal occlusion of the left main. SPECT imaging was false-negative due to balanced ischaemia

Although a number of imaging modalities are available to non-invasively evaluate patients with suspected or known coronary artery disease (CAD), myocardial perfusion scintigraphy (MPS) with single photon emission computed tomography (SPECT) has been the workhorse for this purpose for over decades [1]. Notwithstanding its high sensitivity to detect ischaemia (85–90 %), a caveat of a normal perfusion scintigram is the fact that it can also be compatible with balanced ischaemia due to multi-vessel or left main disease [2]. (Fig. 1). A normal scan must therefore be interpreted with care, and a further refinement in risk stratification is warranted even in the presence of a seemingly reassuring result. In this issue of the Netherlands Heart Journal, Bom et al. report on the prognostic value of a normal MPS during a 2-year follow-up period in 762 patients without a prior history of CAD [3]. The event rate was low (4.2 %) and predominantly driven by revascularisation. Cardiac death and non-fatal myocardial infarction occurred in only nine patients (1.2 %). These results are consistent with pooled analysis from large databases comprising close to 40,000 patients, which yield an annualised event rate of 0.6 % following a normal perfusion SPECT scan [4]. Multivariate analysis by Bom and colleagues revealed that male gender, a positive stress ECG, and a reduced left ventricular (LV) ejection fraction (<45 %) had a negative impact on prognosis. Some other well-documented prognostic risk factors such as age, type of stressor (adenosine vs. exercise), renal failure, and diabetes failed to reach statistical significance, which is probably attributable to the relatively limited sample size of the current study. These data highlight that a normal test result must be interpreted in light of the clinical risk profile of the patient, i.e. the pre-test likelihood of disease. According to Bayes’ theorem, a negative test should alert us when pre-test probability is high as it does not rule out disease as it does when pre-test probability is low.

Besides clinical risk factors, alternative imaging parameters may also aid in further risk stratification of a normal MPS. Transient ischaemic dilation (TID) has been linked to extensive CAD due to post-stress LV dysfunction, as a result of stunning and/or diffuse subendocardial hypoperfusion, which mimics LV enlargement by reduced subendocardial tracer uptake during stress. Abidov et al. explored the prognostic value of TID in patients with a completely normal MPS and identified TID (with an optimal stress-to-rest ratio of 1.21) as an independent prognostic factor with a threefold increase in event rate [5]. In recent years, nuclear imaging has been fused with computed tomography (CT) to facilitate CT-based attenuation correction, but additionally allows to acquire calcium scoring (CAC) and CT coronary angiography (CCTA) within a single scanning session [6]. Combining CAC with myocardial perfusion imaging adds incremental prognostic value in patients with and without myocardial ischaemia, although ischaemia appears to be a more potent predictor of future cardiac events than coronary calcification [7]. Improved risk stratification, by adding anatomical information of CCTA to functional data obtained with MPS, has also been documented by Van Werkhoven et al. [8] Annualised hard event rate of a normal MPS significantly increased when CCTA displayed a coronary lesion of more than 50 % (0.6 vs. 3.8 %).

Another powerful prognostic indicator is quantitative perfusion imaging. As already alluded to in Fig. 1, SPECT is a qualitative imaging technique whereby perfusion defects are identified based on the relative distribution of the tracer. Unfortunately, conditions that are accompanied by lack of normal myocardium to act as a reference limit such a qualitative approach and may yield false-negative results or underestimate the extent of disease (e.g. in case of multivessel disease and/or microvascular dysfunction). Cardiac positron emission tomography (PET), however, is becoming increasingly available and offers the possibility to quantify myocardial blood flow in absolute terms (i.e. ml∙min^−1^∙g^−1^) and calculate coronary flow reserve (CFR) [9]. A number of quantitative cardiac perfusion PET studies have unambiguously demonstrated that a blunted CFR (generally defined <2), in the presence of apparently normal relative myocardial perfusion imaging, is accompanied by an unfavourable prognosis as compared with preserved flow reserve [6]. Moreover, this effect is the strongest predictor for adverse cardiac events in symptomatic patients with normal relative myocardial perfusion imaging, and trumps clinical risk scores as well as calcium scoring [10].

The study by Bom et al. reminds us that a normal MPS is not necessarily accompanied by a benign clinical course and may not always unveil potential serious coronary pathology. Fortunately, advances in imaging techniques enhance the diagnostic and prognostic evaluation of this category of patients. Nonetheless, a pivotal issue remains. These novel insights on the prognostic value of patient characteristics and imaging parameters have yet to be translated into improved patient outcome. At present, it is unclear whether a myocardial perfusion imaging guided treatment strategy in patients with overt ischaemia can improve outcome. This will prove to be even more difficult to ascertain in patients with a normal MPS and, on average, low event rate.
